# Measuring digital capital in Italy

**DOI:** 10.3389/fsoc.2023.1144657

**Published:** 2023-05-19

**Authors:** Felice Addeo, Valentina D'Auria, Angela Delli Paoli, Gabriella Punziano, Massimo Ragnedda, Maria Laura Ruiu

**Affiliations:** ^1^Department of Politic and Communication Sciences, University of Salerno, Fisciano, Italy; ^2^Department of Humanities, Philosophy and Education, University of Salerno, Fisciano, Italy; ^3^Department of Social Sciences, University of Naples–Federico II, Naples, Italy; ^4^Department of Arts, Northumbria University, Newcastle, United Kingdom; ^5^Department of Social Sciences, Northumbria University, Newcastle, United Kingdom

**Keywords:** digital capital, Italy, digital divide, validation, composite index

## Abstract

**Introduction:**

This paper aims to theoretically and empirically investigate the concept of digital capital in the Italian context. Digital Capital can be conceived as independent individual capital whose lack within a population can be a cause of digital inequality. Our paper draws from recent works that have measured the Digital Capital as a combination of digital access and digital competences, and have tested this operational definition through an online survey on a UK sample. The results of such research proved the construct validity of the operational definition, thus showing that Digital Capital could be empirically measured. However, a measurement model needs to be tested and validated over time and in different socio-cultural contexts in order to be refined and strengthened, and eventually disseminated on a large scale.

**Method:**

This is the reason why this paper will show the results of a funded research project (named DigiCapItaly) carried out to test the validity of the Digital Capital measure in a different country, i.e., Italy. The data were collected with an online survey using a representative sample (by age, gender and geographical area) of individuals living in Italy aged 18 years or more. The creation of a composite index to measure Digital Capital followed a two-stage Principal Component Analysis approach.

**Results:**

First, the paper provides a methodological framework for facing challenges and pitfalls in operationalizing and assessing a complex concept in social research. Secondly, results show that Digital Capital operational definition works in Italy as well as in the UK, thus legitimizing its recognition as an independent capital.

## 1. Introduction

The growing use of digital solutions in everyday life offers multiple questions and food for thoughts. Unfortunately, the spread of technology is still not the same in every country, due to the digital divide. Following Hilbert (2015, 2016); Van Deursen and Van Dijk (2015, 2019), digital divide is meant as inequality in access to and use of new digital technologies. In these studies, online and offline inequalities are highly linked and influence each other: inequalities seem to extend also to many other aspects of individuals' offline lives and vice versa (Wei et al., 2011; Ragnedda, 2017). Indeed, digital inequalities are not a strictly geographical concept, only linked to the evolution of broadband infrastructure (Warf, 2001; Crang et al., 2006; Riddlesden and Singleton, 2016). Instead, digital inequalities seem to be linked to other aspects, such as age (Neves et al., 2018), working position (Van Deursen and Van Dijk, 2019), income (Martin and Robinson, 2007; Jung et al., 2010), belonging to specific social groups such as the disabled, racial minority groups (Clark and Gorski, 2001, 2002; Wilson et al., 2003), and many others.

Most of the research in this area have shown strong links between social exclusion and digital engagement, which tend to influence each other: those with fewer economic resources, lower social position, and less cultural capital are also affected by this type of inequality.

However, previous studies on digital divide are very far from analyzing all the implications of the phenomenon. Initial definitions of the concept have focused on purely informational aspects (Hamelink, 2000) or technical and IT aspects (Rojas et al., 2004) and circumscribed it to the possession of technologies (Katz and Aspden, 1997; Hoffman and Novak, 1999; DiMaggio et al., 2001). Despite the limited scope of these studies, they deserve the credit of having underlined how the unequal distribution of resources among the population underpins digital inequalities (Helsper et al., 2015). It is with the extension of the concept of digital divide to the use of digital devices (Hargittai, 2002; Peter and Valkenburg, 2006; Van Dijk, 2006), that the importance of users' knowledge and technological skills begins to be acknowledged. These research streams consider also the set of skills and competencies developed through engagement with IT as a constituent of the digital divide (Hamelink, 2000; Prieur and Savage, 2013). Within this perspective, digital divide has a dual nature based on digital competencies and digital resources.

Thus, digital inequalities are conceived as the consequence of the different accumulation and availability of digital resources, both material (such as technological devices and digital infrastructure) and immaterial (digital skills, problem-solving capability, content-creation capacities, etc.).

From this point of view, they can be understood as another form of capital–the Digital Capital-which is the object of this paper. The paper leverages on the recent work by Ragnedda et al. (2019), who have operationalized the concept of Digital Capital and measured it through an online survey based on a representative sample of UK citizens. The results proved the construct validity of the operational definition, thus showing that Digital Capital could be empirically measured.

By leveraging on these conceptual and empirical definitions of Digital Capital, the paper supports the empirical aim to explore and test the validity of the Digital Capital and measure it for the first time in Italy. Specifically, it aims to validate the operational definition of digital capital used in the UK in Italy and to explore how it is correlated with the socio-economic and socio-demographic variables in such a context.

The paper is organized as below. The next sections (The concept of digital capital-The research context) provide a theoretical overview of the concept of digital capital and the rational for choosing Italy as research context. Sections Research design, research method, sample and data collection, Operational definition and measures, and Results deal with the research design, the operationalization procedures and the statistical results. Finally, in the last section findings are discussed and conclusions are drawn.

## 2. The concept of digital capital

We can distinguish three stages in the research on digital inequalities. The first one focuses merely on the differences in users' access to the internet (Hoffman and Novak, 1999; DiMaggio et al., 2001), on purely informational aspects (Hamelink, 2000) or technical and IT aspects (Rojas et al., 2004). In the second stage, some studies go beyond mere accessibility by recognizing differences in the uses of the Internet according to digital skills and competencies (Peter and Valkenburg, 2006; Van Dijk, 2006). The third stage is well summarized by Ragnedda (2017, 2018), who linked the concept to the tangible and intangible outcomes and the benefits of using technological devices, that is exploitation of the advantages of the Internet and the changes in people's life that could improve their living conditions.

Thus, digital inequalities may be conceived as the consequence of the different availability and accumulation of digital resources, both material (such as technological devices and digital infrastructure) and immaterial (digital skills, problem solving, content-creation, etc.), and the different distribution of benefits that users are able to achieve.

This recognition of the possibility that digital assets-in their technological and capability aspects-are socially valuable and can improve life chances makes it possible to conceptualize them as a distinct form of capital, referred to as digital capital (Ragnedda, 2018). The concept of capital here is meant in Bourdieu's (1983) terms as transcending economic aspects and involving internalized and externalized resources able to produce benefits in other arenas. Although independent, digital capital is strongly intertwined with other types of capital (e.g., economic, social, cultural, etc.) (Ragnedda, 2018). This reinforces the idea of a dual process (offline → online → offline) in which offline inequalities produce digital inequalities, which in turn could reinforce inequalities present in offline contexts (social, political, economic, personal) (Ragnedda, 2017, 2018; Ragnedda et al., 2022a).

In this sense Ragnedda (2018) defines Digital Capital as “a set of internalized abilities and aptitudes” (known also as “digital competencies”) as well as “externalized resources” (also called “digital technology”) “that can be historically accumulated and transferred from one arena to another.” Within this perspective, digital capital contributes to life opportunities enhancement by creating a bridge between online and offline realms. Online activities produce *social benefits* such as opportunities for socialization, for creating weak ties and reinforcing strong ties; *economic benefits* such as opportunities in finding employment and better jobs, in accessing online services, in online shopping; *political benefits* in reinforcing citizenship and participation in deliberative democracy; *personal benefits* contributing to entertainment, fitness and health; *cultural benefits* in enhancing cultural engagement and cultural activities (Ragnedda et al., 2022b).

There are previous attempts to define the concept of Digital Capital (Morgan, 2010; Seale, 2012). For example, Seale (2012) defines it as the technological know-how, the informal time invested in enhancing technological skills, the formal time spent in ICT education, the online social network. She is interested in how and whether digital capital promotes the inclusion of disabled students. Morgan (2010) conceptualizes it as a new literacy for today's students unconnected with print-based literacies. In the majority of cases, the concept of digital capital is used at a firm level to indicate the set of resources of the digital economy (Tapscott et al., 2000; Roberts and Townsend, 2015).

However, these studies are theoretical at hearth. On the contrary, Ragnedda (2018) aims to measure digital capital as a specific and independent form of capital and to construct and validate it with sociodemographic and socioeconomic variables. Our work leverages precisely on the operational definition provided and validated by Ragnedda et al. (2019) which articulates the concept of digital capital into two components: digital access and digital competence.

*Digital access* includes the *digital equipment* (devices used to access the Internet), *connectivity* (quality of access to the internet), the *time spent online* and the *support and training* in using the Internet.

**Figure 1:**
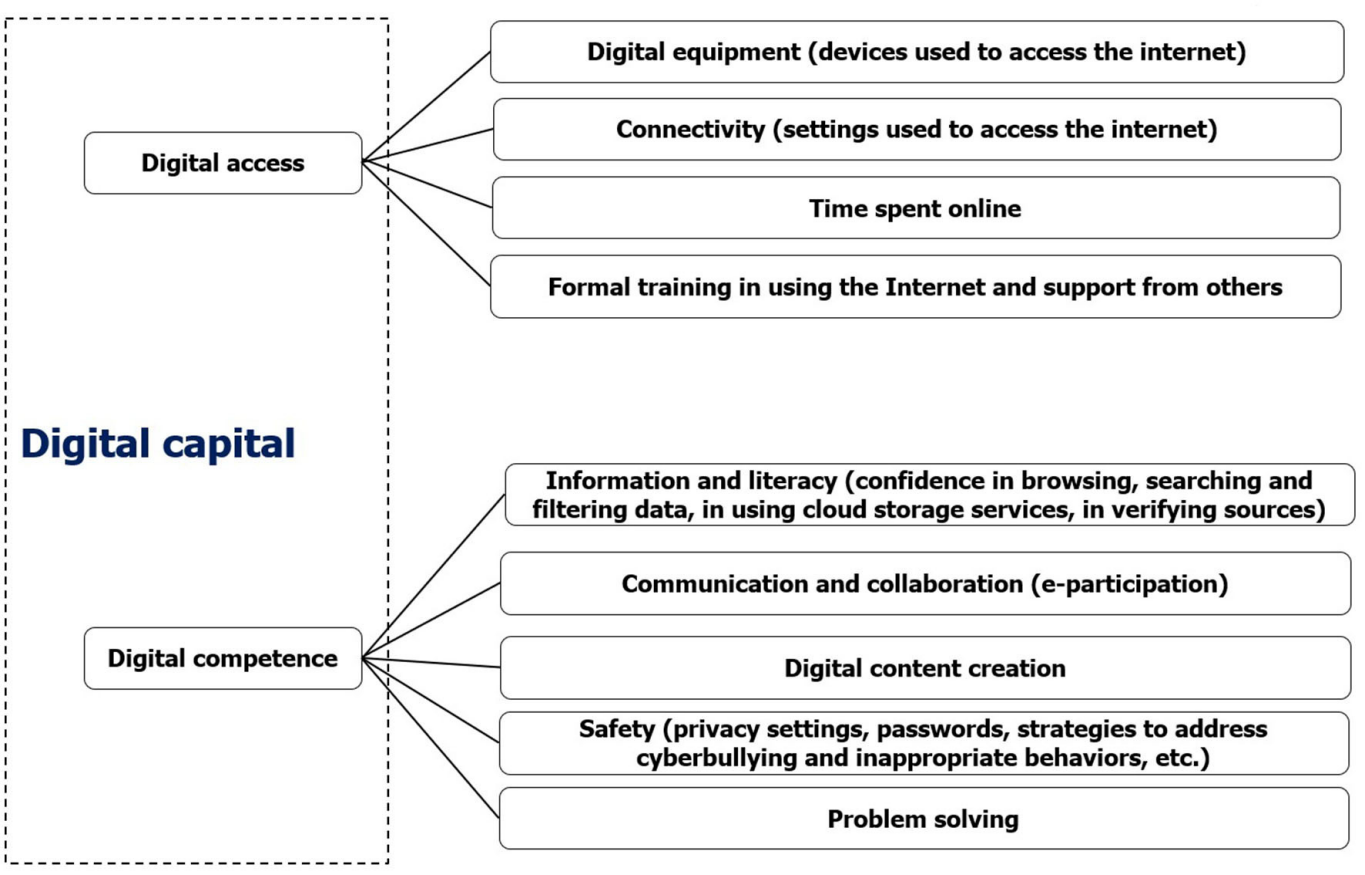
The concept map of digital capital. Source: Authors' own elaboration based on Ragnedda et al. (2019).

*Digital competence* follows the competences framework defined in the European Digital Competence Framework for Citizens (Carretero et al., 2017), so including the individual abilities ranging from the capability in browsing, searching, filtering and verifying information to the capability of creating online content and protect privacy: *information and data literacy, communication and collaboration, digital content creation, safety* and *problem-solving* (see Figure 1).

There are already several studies in the literature that demonstrate how sociodemographic and socioeconomic features are intertwined with online dynamics such as how the Internet is accessed, how well it is managed, the level of engagement in ICT and the breath of online activities (DiMaggio et al., 2004; Zillien and Hargittai, 2009; Robinson et al., 2015). The relationship between sociodemographic and socioeconomic preconditions and online life cannot be considered linear and unidirectional. Instead, it is an interactive and continuously evolving cause-and-effect relationship (Park, 2017). This circular process allows socio-demographic and socioeconomic preconditions to compromise every aspect of online life, starting from online access (Tsatsou, 2011), to digital skills (Jones-Kavalier and Flannigan, 2008) and online activities (Hargittai and Hinnant, 2008; Zickuhr and Smith, 2012).

According to the literature, there are five socio-demographic and socio-economic variables related to Digital Capital:

- Income: As shown in several previous studies (Witte and Mannon, 2010; Talukdar and Gauri, 2011; Mardis, 2013; Ragnedda and Muschert, 2013, 2015; Van Deursen et al., 2017), income is supposed to affect various aspects of online life, in particular the possession of technological devices. For this reason, it should positively affects the level of Digital Capital;
- Age: many studies have found a negative association between age and digital skills, engagement and activities (Lenhart et al., 2008; Lee et al., 2011; Dutton et al., 2013; Blank and Groselj, 2014), so it is assumed that digital capital is negatively related to age;
- Gender: Although in many developed countries with a high Internet penetration, gender digital divide has been reduced or even bridged (Blank and Groselj, 2015;), there are many other developing countries where we can still find a gender gap in terms of frequency, intensity and type of internet use (Wasserman and Richmond-Abbott, 2005; Hargittai, 2010; Haight et al., 2014; Hargittai and Shaw, 2015). Thus, men are supposed to be more likely to have a higher level of Digital Capital;
- Education: Previous studies have shown that the educational level positively influences the level of Digital Capital (Attewell, 2001; Clark and Gorski, 2001, 2002; Mossberger et al., 2007; Shelley, 2009), establishing a positive relationship: users with higher education are more successful in online activities and have better management skills (Van Deursen and Van Dijk, 2013; White and Selwyn, 2013; Blank and Groselj, 2014).
- Place: The literature shows that urbanization influences the level of digital capital. In central areas the presence of infrastructures allows for a greater internet penetration than in rural areas (Crang et al., 2006; Mardis, 2013; Townsend et al., 2013; Ashmore et al., 2015; Philip et al., 2017). This would make geographical differences significant: people from the city (more exposed to technology), are assumed to have a higher level of Digital Capital than those living in peripheral areas.

## 3. The research context

The rationale of a study on Digital Capital in Italy is consequence of the low level of digital skills and knowledge, as testified by the Digital Economy and Society Index (DESI)^1^ developed by the European Commission on an annual basis from 2014. The DESI describes the digital performance and tracks the progress of each member states, with the aim to help them to improve their own weaknesses. The results show significant gaps in both basic and advanced digital skills, in terms of the four dimensions of the DESI Index: *Connectivity* (fixed and mobile broadband coverage), *Human capital* (Internet user skills and advanced skills), *Integration of digital technology* (business digitization and e-commerce), *Digital public services* (e-government).

The DESI 2022 report, based on 2021 data, shows Italy among the poorest performers. Italy ranks 18th out of 27 Member States, with a score of 49.3 out of 80. What seems to be relevant is that only the 46% of the 16–74 years old have at least basic digital skills (54% in the EU) and only 23% have more than basic digital skills (26% in the EU). Generally, the area where greater progress is required is the *Human Capital* dimension, “Internet user skills” and “advanced skills.” In this area Italy tend to be near the bottom of the ranking (Italy is ranked 25th out of 27 European countries). This shows that digital inequalities are much more complex and refer to not only the possession of devices, but also the possibility of developing skills in order to benefit from them. The third dimension of the DESI index, *Digital public services*, also shows major challenges: while most Europeans engage in a wide range of online activities, Italy is ranked 18th out of 27. This shows that once again the Italian position is below the European average (58.5 Italian vs. 67.3 European out of 80). Italy demonstrates to perform better in the *Integration of digital technology* showing a score above the European average (40.7 vs. 36.1 for the European average) and placing 8th out of 27 countries. Among the most interesting results, Italy is in a good position regarding SMEs with at least a basic level of digital intensity (60%, well above the EU average of 55%), and the use of electronic invoicing by enterprises (95% of Italian companies compared to 32% of the European average). However, the use of big data is still low (with an Italian average of 9% compared to 14% at the European level). These mostly positive results are probably related to policy aimed at the digital growth of Italian businesses. Whereby, the growth cannot be considered entirely natural but it is mainly an induced process. Indeed, such policy is included in a more comprehensive five-year plan called “Italia 2025” by the Minister for Technological Innovation and Digitization aimed at a digital transformation of the country. Moreover, one of the most encouraging news about the *Connectivity* dimension is that Italy is one of the most advantaged countries in the 4G replacement project, in accordance with the 5G Action Plan for Europe. On the other hand, the Italian position regarding mobile connectivity is quite controversial: while mobile connectivity coverage is still one of the worst in Europe, Italy is one of the leader countries in mobile only access (with 23% of households compared to 11% in Europe). Results on *Digital public services* also seems controversial: although below the European average (67 vs. 72%), Italy performs well in terms of offering digital and open data services. The low score seems due to the poor interaction both between users and online services and between users and public authorities that should encourage the use of public data and digital services. Once again, there is evidence that the digital culture in Italy is weak in terms of the availability of services and infrastructures.

However, despite the low growth of digitalization progress in the last 5 years, in 2022 Italy has climbed a few places in the European ranking (moving from a DESI index score of 45.5 to 49.3). This small comeback gives hope that the level of digitization in Italy will improve over time, also thanks to scientific and academic research that could draw attention to digital issues.

## 4. Research design, research method, sample and data collection

The main objective of our study is to measure digital capital following the research approach developed by Ragnedda et al. (2019) and implemented in the United Kingdom, with the necessary adaptations for the Italian context. Thus, the paper aims to answer the following research questions.

RQ1: Is it possible to implement and validate the operational definition used in the UK to measure Digital Capital, conceived as a distinct and independent capital, in the Italian context?

RQ2: Does Digital Capital measured in Italy behave in the same way as in the UK? In other words, through a construct validation procedure, has the Digital Capital in Italy similar statistical relationship with the socio-economic and the relevant socio-demographic variables (age, gender, level of education, income, area of residence)?

Our research questions required different statistical techniques to be properly addressed. More specifically, RQ1 was addressed by conducting Exploratory Factor Analysis (EFA) with a two-stage Principal Component Analysis approach (Di Franco and Marradi, 2013), in order to build and validate the Digital Capital Index (DCI). To answer RQ2 and construct validate the DCI, bivariate analysis was carried out using five sociodemographic variables: gender; age; education; income and occupation. Data analysis was carried out using the statistical analysis software, IBM SPSS Statistics 25^®^.

The data were collected on December 2021 through a web survey involving Italian people aged 18 and over. As with the UK study, the research team opted for online-only administration of the questionnaires, as this meets the gnoseological need to include only those who (whether for age reasons or not) have a minimum level of technical competence and access. The questionnaire is composed by four sections with a total of fifty questions. The first section collects sociographic information about the interviewee (such as gender, age, educational qualification, etc.); the second section focuses on information about Internet use (e.g., which devices one usually uses, from which places one generally connects to the Internet, whether one has enrolled in online courses, etc.); the third section delves into the sociocultural and socioeconomic background of the respondent, with a set of questions about different life domains (economic, social, political, cultural, and personal) (e.g., the respondent's the degree of political interest and participation; leisure time occupation; sociocultural status of birth family; types of activities carried out online, etc.); finally, the fourth section deals with life satisfaction (with a focus on economic conditions). The average time required to complete the questionnaire was 25 min. The survey was pre-tested on 20 Internet users in two rounds. Based on the feedback, changes were made.

The sample was built by extracting respondents from an online panel provided by Toluna, a professional organization for market research. The web survey collected 1,100 full responses with a response rate of approximately 8% of contacted people. The sample has been built to be representative of the Italian population with reference to gender, age, and geographical area (as shown in Table 1). The sample size was calculated with a 2.95% margin of error at 95% confidence level.

**Table 1:** Sample-population comparison.

		**Sample**		**Population**
		**Count**	**%**	**%**
Gender	Male	534	48.5	48.3
	Female	566	51.5	51.7
Age	18–29	159	14.4	14.2
	30–44	258	23.5	21.1
	45–65	400	36.4	38.0
	Over 65	283	25.7	26.7
Geographical area	North-West	295	26.8	26.8
	North-East	229	20.8	19.6
	Center	207	18.9	19.9
	South	262	23.8	22.9
	Islands	107	9.7	10.9

Source for comparison: ISTAT (2021).

## 5. Operational definition and measures

Following Ragnedda et al. (2019), the construction of DCI was carried out in three steps: First, a univariate analysis was used to check for the data quality and provide an overview of the results; then, a multivariate analysis was conducted to create the DCI; and finally, a bivariate analysis was conducted to test the validation of the DCI.

The analysis was performed as described in detail below.

To create the DCI, we first built the two sub-indices of Digital Access (by combining the set of digital access' questions shown in Table 2) and Digital Competences (by considering the sub-components shown in Table 3). A two-stage Principal Component Analysis approach was run to develop a Digital Capital Index from Digital Access and Digital Competences indices, able to synthesize a high number of items and to simplify the interpretation of the results (Di Franco and Marradi, 2013). After the first extraction, each factor (and the loading variables) was independently analyzed to remove those variables that were not strictly connected to the concepts under analysis. Considering the Kaiser (1960) criterion of retaining only those factors having eigenvalues of 1 or more, results showed that the extraction of one factor was appropriate to represent the factorial solution; moreover, the average size of the factor loadings (over ± 0.6) is good (Comrey and Lee, 1992), this suggests that all the selected variables contribute to define the factor.

**Table 2:** Digital access: operational definition.

**Sub-component**	**Description**	**Items or modalities**	**Collection**	**Measure**
Digital equipment	Devices used to access the Internet	-Mobile phone or smartphone -Laptop or netbook -Tablet computer -Desktop Computer -Media or game players -Smart Tv -Other devises (e.g., e-book reader, Smartwatch)	Multiple response	Nominal
Connectivity	Quality and Place of access	In which of the following settings do you most frequently access the Internet?	Multiple response	Nominal
Time spent online	First time using the Internet	How old were you when you used the Internet for the very first time?	Open question	Scale
Support and Training	Request for help, formal training received, and help offered	Have you ever had any formal training in using Internet?	Multiple response	Nominal
		If you needed help, would there be someone who could help you with using the Internet?	Closed question	Nominal
		Have you looked or asked for help to use the Internet in the past 3 months?		
		Have you helped someone use the Internet in the past 3 months?		

**Table 3:** Digital competences: operational definition.

**Sub-component**	**Description**	**Items or modalities**	**Collection**	**Measure**
Information and data literacy	Browsing, searching, filtering data, information and digital content	I am confident in browsing, searching and filtering data, information and digital content	Likert Scale -Not at all true of me-Not very true of me-Neither true nor-untrue-Mostly true of me-Very true of me	Scale
	Evaluating data, information and digital content	I regularly verify the sources of the information I find		
	Managing data, information and digital content	I regularly use cloud information storage services or external hard drives to save or store files or content		
Communication and collaboration	Interacting through digital technologies	I actively use a wide range of communication tools (e-mail, chat, SMS, instant messaging, blogs, micro-blogs, social networks) for online communication	Likert Scale -Not at all true of me-Not very true of me-Neither true nor-untrue-Mostly true of me-Very true of me	Scale
	Sharing through digital technologies	I know when and which information I should and should not share online		
	Engaging in citizenship through digital technologies	I actively participate in online spaces and use several online services (e.g., public services, e-banking, online shopping)		
	Managing digital identity	I have developed strategies to address cyberbullying and to identify inappropriate behaviors		
Digital content creation	Developing digital content	I can produce complex digital content in different formats (e.g., images, audio files, text, tables)	Likert Scale -Not at all true of me-Not very true of me-Neither true nor-untrue-Mostly true of me-Very true of me	Scale
	Integrating and re-elaborating digital content	I can apply advanced formatting functions of different tools (e.g., mail merge, merging documents of different formats) to the content I or others have produced		
	Copyright and licenses	I respect copyright and licenses rules and I know how to apply them to digital information and content		
	Programming	I am able to apply advanced settings to some software and programs		
Safety	Protecting devices	I periodically check my privacy setting and update my security programs (e.g., antivirus, firewall) on the device(s) that I use to access the Internet	Likert Scale -Not at all true of me-Not very true of me-Neither true nor-untrue-Mostly true of me-Very true of me	Scale
	Protecting personal data and privacy	I use different passwords to access equipment, devices and digital services		
	Protecting health and wellbeing	I am able to select safe and suitable digital media, which are efficient and cost-effective in comparison to others		
Problem-solving	Solving technical problems	I am able to solve a technical problem or decide what to do when technology does not work	Likert Scale -Not at all true of me-Not very true of me-Neither true nor-untrue-Mostly true of me-Very true of me	Scale
	Identifying needs and technological responses	I can use digital technologies (devices, applications, software or services) to solve (non-technical) problems		
	Creatively using digital technologies	I am able to use varied media to express myself creatively (text, images, audio and video)		
	Identifying digital competence gaps	I frequently update my knowledge on the availability of digital tools		

In the final stage, we adjusted index score to a range from 0 to 100 to simplify its interpretation.

Indeed, to answer the RQ2, we carried out a bivariate analysis between the DCI and socio-demographic variables considered crucial (age, gender, level of education, income, place of residence) to test the validation of DCI. Specifically, we used one-way analysis of variance (ANOVA) to explore the relationships between Digital Capital and gender, income, educational level and place of residence. Meanwhile, we used a correlation analysis to test the statistical relationship between Digital Capital and age. Finally, the relationship between Digital Capital and gender was addressed by performing an independent samples *t*-test.

## 6. Results

### 6.1. RQ1

As mentioned above, the first stage of analysis focused on the building of the Digital Capital Index by combining the Digital Access Index and the Digital Competences Index.

In order to create the Digital Access Index the multiple responses related to each sub-component of Digital Access were conceived as dummy variables and summarized into single variables. The four variables were included in the EFA to test the operational definition and develop the Digital Access Index (see Table 4). The factor scores were saved using the regression method.

**Table 4:** Factor loadings of the variables used for the digital access index.

	**Digital access index**
Digital equipment	0.746
Connectivity	0.756
Time spent online	0.606
Support and training	0.646

Kaiser–Meyer–Olkin (KMO) test = 0.701; Bartlett's test, Sig. < 0.000.

Indeed, the Digital Competence Index was built by directly applying the EFA to the set of items shown in Table 5. The first step of the factor analysis provided a three-factor solution that explained the 58% variance and are named after “Problem-solving,” “Content creation” and “Safety” competencies.

**Table 5:** Factor loadings of the digital competence items.

	**Factors**		
	**Problem solving**	**Content creation**	**Safety**
I am confident in browsing, searching and filtering data, information and digital content			
I regularly use cloud information storage services or external hard drives to save or store files or content		0.553	
I regularly verify the sources of the information I find			
I actively use a wide range of communication tools (e-mail, chat, SMS, instant messaging, blogs, micro-blogs, social networks) for online communication		0.698	
I know when and which information I should and should not share online		0.601	
I actively participate in online spaces and use several online services (e.g., public services, e-banking, online shopping)		0.678	
I have developed strategies to address cyberbullying and to identify inappropriate behaviors		0.501	
I can produce complex digital content in different formats (e.g., images, audio files, text, tables)	0.706		
I can apply advanced formatting functions of different tools (e.g., mail merge, merging documents of different formats) to the content I or others have produced	0.764		
I respect copyright and licenses rules and I know how to apply them to digital information and content			0.632
I am able to apply advanced settings to some software and programs	0.828		
I periodically check my privacy setting and update my security programs (e.g., antivirus, firewall) on the device(s) that I use to access the Internet			0.626
I use different passwords to access equipment, devices and digital services			0.757
I am able to select safe and suitable digital media, which are efficient and cost-effective in comparison to others	0.519		
I am able to solve a technical problem or decide what to do when technology does not work	0.775		
I can use digital technologies (devices, applications, software or services) to solve (non-technical) problems	0.761		
I am able to use varied media to express myself creatively (text, images, audio and video)	0.651		
I frequently update my knowledge on the availability of digital tools	0.712		

Kaiser–Meyer–Olkin (KMO) test = 0.946; Bartlett's test, Sig. < 0.000.

By implementing the two-step factor analysis approach (Di Franco and Marradi, 2013), the three factors were converted into variables by considering only those items with high factor loadings on each component. Then, we performed a second EFA on the three variables, representing “Problem-solving,” “Content creation” and “Safety,” in order to extract a single factor representing Digital Competences (see Table 6). This double step improved the interpretation of the latent dimension by “refining” the results and isolating those features that strongly contribute to the factors.

**Table 6:** Factor loadings of the variables used for the digital access index.

	**Digital competences index**
Problem solving	0.869
Content creation	0.822
Safety	0.866

Kaiser–Meyer–Olkin (KMO) test = 0.707; Bartlett's test, Sig. < 0.000.

The last step of our process was to combine the Digital Access Index and the Digital Competence Index through a further extraction of a single factor representing the DCI (as shown in Table 7). The factor analysis provided a one-factor solution that explained the 72.8% variance and shows that the two components have the same weight in determining Digital Capital.

**Table 7:** Component matrix of the combination between digital access index and the digital competence index.

	**Component 1**
Digital access	0.853
Digital competences	0.853

### 6.2. RQ2

The second research question addressed the validation of digital capital in a different social and cultural context, namely the Italian one. To achieve this, we applied a construct validation procedure in which we tested the DCI with the sociodemographic and socioeconomic variables considered in the literature by previous studies (see Section The concept of digital capital): Education, Age, Gender, Income, and Place of Residence.

Previous studies have shown that education has a positive impact on the digital experience, e.g., levels of access, usage, digital skills, etc. (Clark and Gorski, 2001, 2002; Shelley, 2009; Van Deursen and Van Dijk, 2013; Blank and Groselj, 2014; Ragnedda et al., 2019). Consistent with these findings, the one-way ANOVA shows a positive and statistically significant impact of education on digital capital (see Table 8).

**Table 8:** Relationship between qualification and DCI.

**Qualification**	**Mean**	**Standard dev**.
Some high school, no diploma	43.0	16.9
High school graduate	50.7	14.6
Some college credit, no degree	51.2	13.5
Bachelor's degree	51.9	14.9
Master's degree	60.6	14.7
Postgraduate qualification	62.5	9.5

*F* = 13.692; Sig. < 0.000.

The literature shows that younger people have a higher level of access and use (in terms of digital skills, types of online activities, engagement, etc.) than the older ones (Lee et al., 2011; Dutton and Blank, 2013; Blank and Groselj, 2014; Ragnedda et al., 2019). In this regard, we performed a correlation analysis between age and Digital Capital (shown in Table 9). The correlation coefficient shows a statistically significant negative relationship (−0.404, *p* < 0.000).

**Table 9:** Correlation analysis between age and DCI.

	**Digital capital index**
Age	−0.404^**^

** Correlation is significant at the 0.000 level (two-tailed).

Digital divide literature has shown differences between men and women about digital experience and digital knowledge: several studies show that men are more likely to use digital devices and to develop better digital skills (Ono and Zavodny, 2007; Blank and Groselj, 2014). However, in Ragnedda et al. (2019) this evidence is not supported. Digital gender inequalities are very likely to decrease over the years, especially in more developed societies, as shown by Blank and Groselj (2014). In our analysis, a small gender difference emerged from the one-way ANOVA, with men being slightly better than women, with a positive deviation of 3.6 are. However, the *t-test* (shown in Table 10) returned coefficients about a non-statistically significant relationship (with *F* = 0.025).

**Table 10:** Relationship between gender and DCI.

	**Mean**	**Standard dev**.								
Male	52.8	15.1								
Female	49.2	15.1								
		**Levene's test, f**		***t*** **-test**						
		**F**	**Sign**.	**t**	**gl**	**Sig.(two-tailed)**	**Mean difference**	**Standard error difference**	**95% confidence interval**	
									**Lower**	**Upper**
DCI	Equal variances assumed	0.025	0.873	3.943	1,098	0.000	3.585	0.909	1.801	5.369
	Equal variances assumed			3.943	1094.157	0.000	3.585	0.909	1.801	5.369

Similarly, several scholars (Witte and Mannon, 2010; Talukdar and Gauri, 2011; Ragnedda and Muschert, 2013) have suggested that the level of economic resources can have a major impact on the ability to access digital devices and/or develop specific skills in this regard. In other words, recent literature shows that those who have more resources are also more likely to have access, skills, and engagement related to technology. The results of the one-way ANOVA shown in Table 11 shows a positive relationship between income and DCI, highlighting a 6.3 gap between those with high income and those with low income. However, not only is the difference smaller than expected, but the results are not statistically significant (*F* = 2.995).

**Table 11:** Relationship between income and DCI.

**Income**	**Mean**	**Standard dev**.
<10.000€	48.9	14.3
10.000–20.000€	50.6	14.5
21.000–30.000€	50.9	15.9
31.000–50.000€	52.0	15.1
More than 50.000€	55.2	13.4

F = 2.995; Sig. < 0.018.

This result may shed light on the democratization of technology, due primarily to broader access and use of technology by all segments of the population, but also to the low cost of owning certain digital devices or services that bring technology closer to people.

Geographic location of residence is another important aspect in understanding how access to and use of technology may differ across people. In fact, several works (Ashmore et al., 2015; Philip et al., 2017) show that living in an urban area may have a positive impact on technology experience compared to living in more rural areas. One of the most important aspects in this context is the availability of infrastructure for connectivity, such as network signals, broadband connections, etc. The study by Ragnedda et al. (2019) also contains some scientific evidence along these lines. In this regard, our results can be considered consistent with this research, as Table 12 highlights the relationship between place of residence and DCI, showing a difference of 7.3 between those who live in the center and those who live in rural areas. The results are also statistically significant (with *F* = 18.919 and Sig. < 0.000).

**Table 12:** Relationship between area of residence and DCI.

**Area of residence**	**Mean**	**Standard dev**.
Urban areas	55.1	15.3
Small towns	51.0	14.3
Rural areas	47.8	15.3

F = 18.919; Sig. < 0.000.

## 7. Discussion and conclusion

The aim of this paper was to derive further evidence about the theoretical and empirical validity of the concept of Digital Capital as originally defined and measured in the UK by Ragnedda et al. (2019). As a first step, the operational definition of the concept was adapted to the Italian context. Subsequently, Digital Capital was measured and then tested by comparing it with specific socio-demographic and socio-economic variables.

Actually, the relevant literature shows that digital experience, in terms of access, use, engagement and skills, is closely linked to specific socio-economic factors, such as Education, Age, Gender, Income, and Place of Residence. In other words, Digital Capital, like all other types of capital (economic, political, social, cultural, etc.), is intertwined with and influenced by factors external and internal to individuals and the specific societies in which they live.

In fact, addressing RQ2 enabled us to test the DCI from this point of view; despite its limitations, the bivariate analysis, allowed us to assess the presence and intensity (where possible) of the association between Digital Capital and the socio-demographic and socio-economic variables.

The data analysis provided satisfactory results in line with the reference literature. Among other results, the bivariate analysis showed significant relationships between:

- Qualification and DCI, with a spread of 19.5 between those without an educational qualification and those with a postgraduate qualification, in favor of the latter;
- Age and DCI, showing that young people have a higher level of Digital Capital than older one. This could be justified by the fact that younger people have a better quality of digital experience together with the amount of time spent online than previous generations;
- Area of residence and DCI: unlike the English context, Italy cannot be considered a high internet penetration country due to lack of geographical distributed infrastructures which makes people living in cities more technological advantaged.

However, the bivariate analysis produced not statistically significant in Italy related to:

- Gender and DCI, with a 3.6 non-significant difference between men and women. However, this result is in line with the latest findings in the literature, which show a reduction in the gender technological gap. This is because, especially in more emancipated societies, women like men are exposed to technology on a daily basis;
- Income and DCI. A key to understanding the non-significance of this relationship could be related to the widespread use of digital technologies in the Italian population, beyond the socio-economic class they belong to.

This study demonstrated how Digital Capital is intertwined with the “traditional axes” of social inequalities (particularly education).

Italy presents similarities with UK (they are both countries belonging to Western Europe) but also differences (see section The research context) particularly with reference to internet penetration. It cannot be considered a high internet penetration country as the UK due to territorial imbalances in the broadband accessibility. This consideration help us understanding the main difference between Italian and UK samples: the average of digital capital's score is higher in UK (72.8) than in Italy (51.1). This makes the results particularly meaningful, because we have proved the validity of digital capital measure in two different contexts, thus supporting its operational definition given in previous studies and legitimizes its recognition as a specific and independent form of capital.

However, it would be important and equally interesting to test this tool in other contexts, both in Western Europe and in other parts of the world, both in developed and developing countries. Therefore, cross-national studies are needed to examine the level of digital capital and related outcomes. Such studies also seem highly relevant for understanding and addressing inequalities at the policy level. Indeed, digital capital can be viewed as bridging capital, as it is influenced by pre-existing offline capital (helping to improve individuals' digital skills, capabilities, and access) and influences offline capital by producing different types of benefits (translating Internet experiences into new life opportunities). Because of the close linkages between digital capital and other types of capital (e.g., economic, social, and cultural capital), inequalities in access to and use of the Internet may exacerbate inequalities in other important areas. Conversely, then, by improving the level of individuals' digital capital, it would also be possible to reduce inequalities in tangible outcomes. From this perspective, the introduction of a digital capital index could be an important policy-making and monitoring tool. Looking at so many indicators simultaneously can be challenging when it comes to providing guidance for action. Combining indicators into indices at different levels and into a single composite measure helps to achieve a comprehensive assessment of digital capital, provide a methodology for monitoring over the years, able to indicate the areas at risk of digital divide, the success and failure of national policy initiatives and measures, etc.

However, this research is not without limitations. The use of bivariate analysis provided basic results, which could be further investigated through more comprehensive multivariate analysis to better clarify the relationship between DCI and all the variables tested, also including other factors that might influence this link. In addition to this, this study may represent the basis to develop and validate new methodological instruments to assess extent and quality of digital capital based on the same constructs validated in this paper, such as a multi-item scale based mainly on individual's own descriptions. This will be a direction for further research.

## Data availability statement

The raw data supporting the conclusions of this article will be made available by the authors, without undue reservation.

## Ethics statement

Ethical review and approval was not required for the study on human participants in accordance with the local legislation and institutional requirements. Written informed consent for participation was not required for this study in accordance with the national legislation and the institutional requirements.

## Author contributions

All authors listed have made a substantial, direct, and intellectual contribution to the work and approved it for publication.

## Footnotes

^1^For information and details refer to https://ec.europa.eu/digital-single-market/en/desi.
