# Supplementary material for: Measuring digital capital in Italy
Source: Front Sociol. 2023 May 19;8:1144657. doi: 10.3389/fsoc.2023.1144657 (PMC10235697; doi:10.3389/fsoc.2023.1144657)
Supplement: Supplementary file 1 [file Data_Sheet_1.PDF]

Sezione 1: Informazioni personali

Q1. Genere

1. M 2. F 3. Altro specificare (…………………………) 4. Preferisco non rispondere

Q2. Titolo di studio

- ❑ Nessun titolo di studio;
- ❑ Licenza media;
- ❑ Diploma;
- ❑ Frequenza corsi Università, senza laurea;
- ❑ Laurea triennale;
- ❑ Laurea magistrale
- ❑ Master;
- ❑ Dottorato;
- ❑ Altro specificare (…………………………)

Q3. Età (in anni): …..

Q4. Stato civile

- ❑ Single
- ❑ Convivente di fatto
- ❑ Coniugato/a
- ❑ Separato/a
- ❑ Divorziato/a
- ❑ Vedovo/a

Q5. Attualmente vivi con… (può selezionare più di una risposta)

- ❑ Vivo da solo
- ❑ Il mio/la mia partner
- ❑ Il mio figlio/La mia figlia/I miei figli
- ❑ Mia madre
- ❑ Mio padre
- ❑ Parenti stretti (fratello, sorella)
- ❑ Altri parenti (zii, nonni)
- ❑ Amici
- ❑ Conoscenti / coinquilini

Q6. Condizione occupazionale attuale?

- ❑ Impiegato a tempo pieno (più di 30 ore a settimana)
- ❑ Impiegato part-time (meno di 30 ore a settimana)
- ❑ Disoccupato e/o in cerca di lavoro
- ❑ Lavoratore autonomo, a tempo pieno o part-time
- ❑ In pensione
- ❑ Inserito in un progetto statale di tirocinio (Apprendista/Tirocinante)
- ❑ Casalinga
- ❑ Studente/ssa
- ❑ Altro specificare (…………………………)

Q7. Puoi indicarci la tua attuale posizione? (Chiedi se Q6= 1,2,4)

- ❑ Professioni esecutive (ad esempio: operaio non specializzato, usciere, facchino, bidello, cameriere, commesso, manovale, ecc.)
- ❑ Operaio specializzato
- ❑ Impiegato, intermedio; professioni tecniche (ad esempio: perito elettronico, disegnatore industriale, allenatore, ecc.), del commercio e dei servizi (ad esempio: cuoco, infermiere, ecc.)
- ❑ Quadro, funzionario, ricercatore, insegnante

- ❑ Imprenditore, dirigente, dipendente apicale (ad esempio: ingegnere, medico, professore universitario, ecc.)
- ❑ Libero professionista, lavoratore in proprio, artigiano
- ❑ Altro specificare (…………………………)

Q8. A quale dei seguenti settori appartiene l'azienda per cui lavori? (Chiedi se Q6= 1,2,4)

- ❑ Industria
- ❑ Pubblica Amministrazione (amministrazioni statali, università, scuole, ospedali, enti locali, etc.)
- ❑ Artigianato
- ❑ Agricoltura
- ❑ Credito, assicurazione e tribute
- ❑ Terziario (commercio, servizi, professioni, arti, no profit)
- ❑ Altro specificare (…………………………)

Q9. Dove vivi attualmente?

Provincia ____________
Comune _____________

Q10. Attualmente vivi in…

- ❑ Centro
- ❑ Periferia
- ❑ Area rurale
- ❑ Non sono sicuro
- ❑ Altro specificare (…………………………)

Sezione 2. L'utilizzo di Internet

Q11. Quali dispositivi usi per navigare in Internet? (può selezionare più di una risposta)

- ❑ Cellulare o smartphone
- ❑ Laptop o netbook
- ❑ Tablet computer
- ❑ Desktop Computer
- ❑ Lettori multimediali o console (videogioco)
- ❑ Smart Tv
- ❑ Altri dispositivi (es. e-book reader, Smartwatch)

Q.12. In quali dei seguenti contesti accedi ad Internet più frequentemente? (può selezionare più di una risposta)

- ❑ In biblioteca
- ❑ A casa
- ❑ A casa di amici
- ❑ A scuola/Università
- ❑ Al lavoro
- ❑ Al bar/ internet cafè
- ❑ In tutte le zone Wi-Fi Free
- ❑ Altro (da specificare)

Q.13. Ha mai frequentato un corso per…

	Sì	No	Non ricordo
Acquisire conoscenze informatiche di base (ad es. sulle componenti software e hardware)			

Imparare a utilizzare il pacchetto Office (Word, PowerPoint, Excel, ecc.) o software simili			
Imparare a gestire diversi sistemi operativi (Windows, Android, Linux, iOS, ecc.)			
Acquisire conoscenze ad hoc nel settore del web marketing (SEO, SEM, Web Analytics, Inbound Marketing, ecc.)			
Acquisire conoscenze su aspetti visivi e grafici della comunicazione (con programmi come Photoshop, Lightroom, Illustrator, ecc.)			
Imparare linguaggi di programmazione come PHP, Java, SQL, HTML, ecc.			
Imparare a creare videogame con varie piattaforme			

Q.14. A quanti anni hai avuto accesso ad Internet per la prima volta? ________________________

Q.15. Se avessi bisogno di aiuto, ci sarebbe qualcuno che potrebbe aiutarti ad usare le nuove tecnologie?

- ❑ Sì
- ❑ Forse
- ❑ No

[CHIEDERE SE Q15=Si o Forse]

Q16. Quali delle seguenti persone potrebbe aiutarla con l'uso di internet?

- ❑ Amici;
- ❑ Compagno/a;
- ❑ Parenti;
- ❑ Colleghi di lavoro
- ❑ Compagni di classe;
- ❑ Dipendenti di uffici pubblici;
- ❑ Personale di un negozio di informatica;
- ❑ Help desk;
- ❑ Membri di forum o blog online;
- ❑ Altre persone

Q17. Hai mai cercato o chiesto aiuto per l'utilizzo di Internet negli ultimi tre mesi?

- ❑ Sì
- ❑ No
- ❑ Non ricordo

Q18. Hai mai aiutato qualcuno con l'utilizzo di internet negli ultimi tre mesi?

- ❑ Sì
- ❑ No
- ❑ Non ricordo

[CHIEDERE SE Q18=Si]

Q19. Quali delle seguenti persone hai aiutato?

- ❑ Amici;
- ❑ Compagno/a;
- ❑ Parenti;
- ❑ Colleghi di lavoro
- ❑ Compagni di classe;
- ❑ Membri di forum o blog online;
- ❑ Altre persone

Q20. Quale dei seguenti social network usi maggiormente? (Una risposta per riga)

	Mai	Quasi mai	A volte	Spesso	Molto Spesso
Facebook					
Instagram					
WhatsApp					
Telegram					
Snapchat					
Twitter					
Linkedin					
Youtube					
Pinterest					
Twitch					
TikTok					
Altro specificare					

Q20A. Quali "Altri" social network usa? (Se ha risposto Altro alla Q20) ______________________

Q21. Quanto spesso utilizzi internet per…? (Una risposta per riga)

	Mai	Quasi mai	A volte	Spesso	Molto Spesso
Fare nuove conoscenze					
Cercare o candidarsi per un lavoro					
Esercitarsi con una nuova lingua					
Usare Social Media					
Restare in contatto con amici					
Restare in contatto con la famiglia					
Acquistare prodotti e servizi					
Giocare					
Pagare bollette					
Restare aggiornato sugli ultimi eventi					
Organizzare viaggi					
Lavoro / Affari					
Studiare					
Scaricare o ascoltare musica, ecc (es. iTunes, Spotify)					
Guardare film (es. Netflix, Amazon Prime)					
Usare servizi di streaming online (es. canali TV Rai Play, Mediaset Play, ecc.)					
Partecipare a discussioni politiche					

Q21A. Per quali altre attività utilizza Internet? (Se ha risposto Altro alla Q21)________________

Q22. In che misura sei d'accordo o in disaccordo con la seguente dichiarazione? Utilizzo Internet perchè… (Una risposta per riga)

	Fortemente in disaccordo	In disaccordo	Indeciso	D'Accordo	Fortemente in accordo	Non so
È un modo divertente per trascorrere il tempo						

Mi aiuta a rimanere aggiornato su eventi in tutto il mondo e ad avere informazioni preziose su ogni cosa						
Mi permette di restare in contatto con familiari e amici						
Mi aiuta con gli studi e con il lavoro						
Mi permette di condividere le mie idee e i miei pensieri con amici						
Mi permette di condividere le mie idee e i miei pensieri con persone sconosciute						

Q23. In che misura sei d’accordo o in disaccordo con le seguenti affermazioni? Utilizzo Internet perchè… (Una risposta per riga)

	Fortemente in disaccordo	In disaccordo	Indeciso	D’Accordo	Fortemente in accordo	Non so
La mia famiglia e i miei amici mi incoraggiano a utilizzare strumenti tecnologici come Internet e cellulari						
Al giorno d’oggi, bisogna essere sempre connessi. Tutti lo sono						
Quello che pubblico online (post, foto, eventi) può avere un impatto sul mio futuro						
Se non resto al passo con lo sviluppo delle tecnologie mi sento indietro rispetto agli altri						
Saper usare la tecnologia è un beneficio per le mie attività quotidiane						

Q24. Indica quanto le seguenti affermazioni sull’uso di internet sono accurate per te (Una risposta per riga)

	Non è affatto vero	Non è del tutto vero	Non è nè vero nè falso	Per lo più vero	Molto vero	Non lo so e/o Non applicabile
Mi sento sicuro quando navigo online per cercare informazioni e contenuti digitali						
Utilizzo regolarmente i servizi di archiviazione delle informazioni su cloud o dischi rigidi esterni per salvare o archiviare file o contenuti						
Verifico abitualmente le fonti delle informazioni che trovo						

Uso molti strumenti per comunicare online (e-mail, chat, SMS, messaggistica istantanea, blog, micro-blog, social network)						
So perfettamente quando e quali informazioni è il caso di condividere online						
Partecipo attivamente a spazi online e utilizzo diversi servizi online (ad es. Servizi pubblici, e-banking, acquisti online, ecc.)						
Ho sviluppato strategie per contrastare il cyberbullismo e identificare comportamenti inappropriati						
Posso produrre contenuti digitali complessi in diversi formati (ad esempio immagini, file audio, testo, tabelle)						
Sono in grado di applicare funzioni di formattazione avanzate con diversi strumenti (ad esempio l'invio di e-mail di massa – mail merge; l'unione di documenti di differenti formati., etc.) ai contenuti prodotti da me o da altri						

Q25. Indica quanto le seguenti affermazioni sull'uso di internet sono accurate per te (Una risposta per riga)

	Non è affatto vero	Non è del tutto vero	Non è nè vero nè falso	Per lo più vero	Molto vero	Non lo so e/o Non applicabile
Rispetto le regole sul copyright e sui diritti d'autore e so come applicarle alle informazioni e ai contenuti digitali						
Sono in grado di applicare impostazioni avanzate ad alcuni software e programmi						
Controllo abitualmente le mie impostazioni sulla privacy e aggiorno i miei programmi di sicurezza (ad esempio antivirus, firewall) sui dispositivi che uso per accedere a Internet						
Uso password diverse per accedere a dispositive e servizi digitali						
Sono in grado di scegliere media digitali sicuri e adatti, che sono più efficienti e convenienti di altri						
Sono in grado di risolvere un problema tecnico o decidere cosa fare quando uno strumento digitale non funziona						

Posso usare le tecnologie digitali (dispositivi, applicazioni, software o servizi) per risolvere problemi (non tecnici)						
Sono in grado di utilizzare vari media per esprimermi in modo creativo (testo, immagini, audio e video)						
Aggiorno abitualmente la mia conoscenza sulla disponibilità di strumenti digitali						

Q26. Di seguito ti verranno proposte una serie di affermazioni riguardo le tue esperienze online. Rispondi indicando quanto ritieni siano accurate a descriverti. Ti chiediamo di indicare da 0 a 10 (dove 0 significa "Per niente accurate" e 10 "Assolutamente accurate").

	0	1	2	3	4	5	6	7	8	9	10
So usare molti dispositivi per connettermi ad Internet											
Non importa il luogo dove sono, trovo sempre il modo di connettermi ad Internet											
Uso internet da molto tempo											
Quando gli altri hanno un problema con l'uso di Internet si rivolgono a me											
So muovermi nella rete sapendo su quali fonti fare affidamento											
Nel corso della mia esperienza online, ho imparato a condividere i miei pensieri attraverso diversi dispositivi e piattaforme di comunicazione											
Nel corso della mia esperienza online, ho imparato a usare diversi strumenti per creare contenuti testuali e audiovisivi											
Sono in grado di scegliere la modalità più appropriata per proteggere i miei dati personali (ad es. indirizzo, numero di telefono, password).											
Quando ho un problema tecnico con un dispositivo digitale, so sempre come muovermi per risolverlo											

Sezione 3. Background socio-culturale

Q27. Puoi indicarmi il tuo grado di accordo con le seguenti affermazioni?

	Per niente d'accordo	Poco d'accordo	Né in Accordo/ Né in Disaccordo	Abbastanza d'accordo	Molto d'accordo
Gran parte della gente è degna di fiducia					
Non si è mai sufficientemente prudenti nel trattare con la gente					
La gente, in genere, guarda prevalentemente al proprio interesse					
Gli altri, se gli si presentasse l'occasione, approfitterebbero della mia buona fede					
Ritengo che gli altri siano, nei miei confronti, sempre corretti					

Q28. Ora ti elenchiamo una serie di associazioni. Spunta quelle a cui sei iscritto o sei stato iscritto in passato.

	Non ho mai partecipato	Ho partecipato in passato ma non attualmente	Partecipo attualmente come membro (senza ruoli decisionali)	Partecipo attualmente con ruoli decisionali
Associazioni ricreative (sportive o hobbistiche)				

Associazioni religiose (es enti cattolici, gruppi parrocchiali o equivalenti in altre religioni)				
Associazioni culturali (artistiche o di promozione culturale)				
Associazioni ambientali				
Associazioni umanitarie (volontariato, assistenza, tutela diritti umani)				
Associazioni di promozione sociale (APS)				
Associazioni territoriali (comitati di quartiere)				
Associazioni politiche (partiti, sindacati, movimenti)				
Associazioni di professionisti o di categoria				
Associazioni di consumatori				
Altri gruppi specificare (______________)				

Q29. Quanto spesso frequenti i seguenti gruppi di persone?

	Mai	Quasi mai	Raramente	A volte	Spesso	Molto Spesso
Vicini e abitanti del comune in cui vivo						
Colleghi di lavoro e di studio attuali						
Vecchi colleghi e compagni						
Amici di infanzia						
Amici dell'associazione						
Altri gruppi (parrocchia, sport, ecc…)						

Q30. Ora ti elenchiamo alcune cose che la gente fa per partecipare alla vita politica e sociale. Per ciascuna delle seguenti attività, rispondi "Sì" se ti è capitato di farla negli ultimi due anni; altrimenti rispondi "No".

	Sì	No
Partecipare a cortei, manifestazioni, scioperi o assemblee		
Sostenere forme di finanziamento etico (raccolta fondi per scopi di solidarietà o beneficienza)		
Firmare una petizione pubblica o un referendum		
Lavorare per risolvere un problema del tuo quartiere o paese		
Partecipare a campagne elettorali		
Acquistare o rifiutare di acquistare un prodotto per motivi politici, etici o ambientali (boicottaggio)		
Interrompere un servizio pubblico per protesta o occupare luoghi pubblici o fabbriche		
Inviare email o post sui social media per comunicare con rappresentanti politici e PA		
Segnalare disservizi e suggerire proposte per migliorare servizi pubblici		
Pubblicare contenuti su blog, gruppi e forum istituzionali, politici, culturali e di informazione		
Organizzare proteste in rete (mailbombing, netstrike, etc.)		

Q31. Con quale frequenza
[una risposta per riga]

	Mai	Meno di 1 giorno alla settimana	1 o 2 giorni alla settimana	3 o 4 giorni alla settimana	Tutti i giorni o quasi
Ascolti le notizie politiche al TG					
Segui le notizie politiche sui social media					
Discuti di politica con amici e/o famigliari					

Leggi quotidiani					
Leggi libri					

Q32. Hai votato?

- ❑ Nelle ultime elezioni politiche nazionali
- ❑ Nelle ultime elezioni amministrative (regionali, comunali)
- ❑ Non ha votato per nessuna delle due elezioni
- ❑ Non ha requisiti per votare
- ❑ Preferisco non rispondere

Q33. Quanto spesso hai... (Una risposta per riga)

	Mai	Quasi mai	A volte	Spesso	Molto Spesso
Sfogliato riviste/articoli di stile e moda					
Cercato informazioni su come migliorare la sua forma fisica					
Utilizzato programmi di allenamento o di nutrizione					
Chiesto un consiglio su una condizione medica					
Cercato informazioni o chiesto opinioni ad altri per comprendere problemi o temi che le interessano					

Q34. Negli ultimi 3 mesi della tua esperienza online, quanto spesso hai… (Una risposta per riga)

	Mai	Quasi mai	A volte	Spesso	Molto Spesso
Cercato le ultime notizie su temi di attualità					
Cercato le ultime notizie di sport					
Cercato informazioni sulla tua cultura e le tue tradizioni					
Cercato informazioni su corsi di formazione, certificazioni o enti che rilasciano certificati					
Interagito con persone che appartengono a diverse etnie					
Contattato persone che condividono la loro fede religiosa o spirituale					

Q35. Pensa alla tua vita quotidiana prima dell’attuale pandemia da Covid-19. Con quale frequenza ti è capitato di svolgere le seguenti attività?

	Mai	Quasi mai	A volte	Spesso	Molto spesso
Ascoltare musica					
Leggere					
Andare al cinema o assistere a una proiezione di film all'aperto					
Visitare musei o teatri					
Assistere a concerti					
Imparare altre lingue					
Praticare arti e attività artigianali					
Uscire con amici					
Viaggiare					
Giocare ai videogiochi					
Guardare sport in TV					
Ascoltare musica classica					

Praticare sport					

Q36. Qual è l'ultimo titolo di studio conseguito da tuo padre?

- ❑ Nessun titolo
- ❑ Licenza elementare
- ❑ Licenza media;
- ❑ Diploma;
- ❑ Laurea;
- ❑ Post-Laurea (Master, dottorato, ecc.)

Q37. Quale è la condizione occupazionale di tuo padre?

- ❑ Legislatori, imprenditori e alta dirigenza
- ❑ Professioni intellettuali, scientifiche e di elevata specializzazione
- ❑ Professioni tecniche
- ❑ Professioni esecutive nel lavoro d'ufficio
- ❑ Professioni qualificate nelle attivita' commerciali e nei servizi
- ❑ Artigiani, operai specializzati e agricoltori
- ❑ Conduttori di impianti, operai di macchinari fissi e mobili e conducenti di veicoli
- ❑ Professioni non qualificate
- ❑ Forze armate

Q38. Qual è l'ultimo titolo di studio conseguito da tua madre?

- ❑ Nessun titolo
- ❑ Licenza elementare
- ❑ Licenza media;
- ❑ Diploma;
- ❑ Laurea;
- ❑ Post-Laurea (Master, dottorato, ecc.)

Q39. Quale è la condizione occupazionale di tua madre?

- ❑ Legislatori, imprenditori e alta dirigenza
- ❑ Professioni intellettuali, scientifiche e di elevata specializzazione
- ❑ Professioni tecniche
- ❑ Professioni esecutive nel lavoro d'ufficio
- ❑ Professioni qualificate nelle attività commerciali e nei servizi
- ❑ Artigiani, operai specializzati e agricoltori
- ❑ Conduttori di impianti, operai di macchinari fissi e mobili e conducenti di veicoli
- ❑ Professioni non qualificate
- ❑ Forze armate

Q40. Pensando alle sue attività online negli ultimi 12 mesi, quanto sei d'accordo o in disaccordo con ognuna delle seguenti affermazioni? Internet ha migliorato le mie capacità di … (UNA RISPOSTA PER RIGA)

	Fortemente in disaccordo	In disaccordo	Indeciso	D'Accordo	Fortemente in accordo	Non so
Cercare informazioni su servizi pubblici nazionali						
Cercare informazioni su un deputato, consigliere comunale, partito o candidato politico						
Chiedere consigli su servizi pubblici a un rappresentante di un'istituzione pubblica						
Organizzare una protesta						
Lanciare o firmare una petizione						

Q41. Pensando alle sue attività online negli ultimi 12 mesi, quanto è d'accordo o in disaccordo con ognuna delle seguenti dichiarazioni? Internet ha migliorato le mie capacità di… (UNA RISPOSTA PER RIGA)

	Fortemente in disaccordo	In disaccordo	Indeciso	D'Accordo	Fortemente in accordo	Non so
Vendere qualcosa di mio						
Allargare la mia attività economica/commerciale						
Cercare informazioni su polizze assicurative						
Cercare informazioni su tassi di interesse						
Cercare un lavoro migliore						
Trovare informazioni su prezzi di prodotti o servizi						
Comparare diversi prodotti o servizi						

Q42. Pensando alle tue attività online negli ultimi 12 mesi, quanto sei d'accordo o in disaccordo con ognuna delle seguenti affermazioni? Internet ha migliorato le mie capacità di…
(UNA RISPOSTA PER RIGA)

	Fortemente in disaccordo	In disaccordo	Indeciso	D'Accordo	Fortemente in accordo	Non so
Trovare un corso o un ente che fornisce corsi						
Interagire con/ Capire altre culture						
Controllare le opinioni degli altri su un corso o un luogo per studiare						
Imparare o adoperare nuove lingue						
Leggere nuovi libri o articoli						
Organizzare le vacanze o un viaggio						
Approfondire/Migliorare le mie conoscenze musicali						

Q43. Pensando alle tue attività online negli ultimi 12 mesi, quanto sei d'accordo o in disaccordo con ognuna delle seguenti affermazioni? Internet ha migliorato le mie capacità di….(UNA RISPOSTA PER RIGA)

	Fortemente in disaccordo	In disaccordo	Indeciso	D'Accordo	Fortemente in accordo	Non so
Restare in contatto con familiari che vivono lontano						
Restare in contatto con amici che vivono lontano						
Allargare la mia rete e incontrare nuovi amici						
Cercare informazioni su attività commerciali						
Interagire con persone che condividono i miei stessi interessi e hobby						
Discutere questioni sociali						

Q44. Pensando alle tue attività online negli ultimi 12 mesi, quanto sei d'accordo o in disaccordo con ognuna delle seguenti affermazioni? Internet ha migliorato le mie capacità di…
(UNA RISPOSTA PER RIGA)

	Fortemente in disaccordo	In disaccordo	Indeciso	D'Accordo	Fortemente in accordo	Non so
Migliorare e/o cambiare il mio stile di vita						
Migliorare la mia forma fisica						
Chiedere ad altri informazioni su programmi di formazione						
Migliorare la mia comprensione di problemi e questioni che mi interessano						
Conoscere opinioni altrui su problemi e questioni che mi interessano						

Sezione 4. Soddisfazione generale

Q45. Soddisfazione generale

In generale, quanto è soddisfatto della sua vita oggi? Dove 0 è "per niente soddisfatto" e 10 è "completamente soddisfatto". (UNA RISPOSTA PER RIGA)

	0	1	2	3	4	5	6	7	8	9	10
Relazione amorosa											
Situazione finanziaria della famiglia											
Amicizia											
Carriera/ Opportunità lavorative											
Educazione											
Vita sociale											
Relazioni familiari											
Relazioni di quartiere/con i miei vicini di casa											

Finanze
Si ritiene che la ricchezza sia un mezzo importante per esercitare potere e influenza nella società, ma sono necessarie ulteriori ricerche per comprenderne i meccanismi. Ti preghiamo di dirci qualcosa sulla tua condizione economica (questi dati saranno archiviati in modo sicuro, in forma anonima e non saranno condivisi con nessun altro).

Q46. Qual è il tuo reddito familiare netto annuale (tasse escluse)?
Reddito totale del nucleo famigliare: personale, del coniuge/di altri membri della famiglia (partner?)
- ❑ Meno di 10.000€
- ❑ 10.000€ - 20.000€
- ❑ 21.000€ - 30.000€
- ❑ 31.000€ - 50.000€
- ❑ 51.000€ - 70.000€
- ❑ 71.000€ - 100.000€
- ❑ Più di 100.000€

Q47. Qual è il valore della sua abitazione di proprietà o in affitto?
(Valore di tutte le proprietà possedute da te o dal tuo partner o altri significativi)
1. Di proprietà

- ❑ Meno di 125.000€
- ❑ 126.000€ - 250.000€
- ❑ 251.000€ - 500.000€
- ❑ Più di 500.000€

2. In affitto

- ❑ Meno di 125.000€
- ❑ 126.000€ - 250.000€
- ❑ 1.3. 251.000€ - 500.000€
- ❑ 1.4.Più di 500.000€

Q48. Quante stanze ha la casa nella quale vivi? _________

Q49. Possiedi qualche tipo di risparmio?
Pensione, azioni, investimenti etc.
- ❑ Nessuno
- ❑ €0 – 10.000€
- ❑ 11.000€ - 25.000€
- ❑ 26.000€ - 50.000€
- ❑ 51.000€ - 100.000€
- ❑ Più di 100.000€

Q50. Nel prossimo mese le mie entrate…
- ❑ Non mi permetteranno di coprire i miei fabbisogni
- ❑ Mi consentiranno di vivere dignitosamente
- ❑ Mi consentiranno di condurre una vita agiata
