# Supplementary material for: Measuring digital capital in Italy
Source: Front Sociol. 2023 May 19;8:1144657. doi: 10.3389/fsoc.2023.1144657 (PMC10235697; doi:10.3389/fsoc.2023.1144657)
Supplement: Supplementary file 2 [file Table_1.DOCX]

Supplementary Material

(descriptive statistics not to be included in the paper)

**Measuring Digital Capital in Italy**

Felice Addeo^1*^, Valentina D’Auria^1^, Angela Delli Paoli^2^, Gabriella Punziano^3^, Massimo Ragnedda^4^, Maria Laura Ruiu ^5^

^1^University of Salerno, Department of Politic and Communication Sciences, Italy

^2^Universityof Salerno, Department of Humanities, Philosophy and Education, Italy

^3^ University of Naples – Federico II, Department of Social Sciences, Italy

^4^ Northumbria University, Department of Arts, Newcastle, UK

^5^ Northumbria University, Department of Social Sciences, Newcastle, UK

**^*^Felice Addeo:**

Corresponding Author

faddeo@unisa.it

**Descriptive statistics - Digital Access**

| Devices used to access the Internet | count | % |
| --- | --- | --- |
| Mobile phone or smartphone | 1046 | 95.1 |
| Laptop or netbook | 533 | 48.5 |
| Tablet computer | 517 | 47.0 |
| Desktop Computer | 575 | 52.3 |
| Media or game players | 84 | 7.6 |
| Smart Tv | 406 | 36.9 |
| Other devices (e.g. e-book reader, Smartwatch) | 44 | 4.0 |

Table 1. Devices used to access the Internet

| In which of the following settings do you most frequently access the Internet? | count | % |
| --- | --- | --- |
| Library or other setting open to the public | 46 | 4.2 |
| At home | 1084 | 98.5 |
| At a friend’s home | 160 | 14.5 |
| At school | 86 | 7.8 |
| At work | 443 | 40.3 |
| Café | 132 | 12.0 |
| Free Wi-Fi anywhere | 162 | 14.7 |

Table 2. Quality and Place of access

| How old were you when you used the Internet for the very first time? | count | % | cumulative % |
| --- | --- | --- | --- |
| 1 to 10 years | 63 | 5.7 | 5.7 |
| 11 to 20 years | 408 | 37.1 | 42.9 |
| 21 to 30 years | 215 | 19.6 | 62.4 |
| 31 to 40 years | 190 | 17.3 | 79.7 |
| 41 to 50 years | 145 | 13.2 | 92.9 |
| 51 to 60 years | 48 | 4.4 | 97.4 |
| 61 to 70 years | 20 | 1.8 | 99.2 |
| Over 70 years | 6 | 0.6 | 100 |

Table 3. First time using the Internet

| Did you ever attended a course for… | count | % |
| --- | --- | --- |
| Acquire basic computer knowledge (e.g. about software and hardware components) | 546 | 49.6 |
| Learning to use the Office package (Word, PowerPoint, Excel, etc.) or similar software | 593 | 53.9 |
| Learning to manage different operating systems (Windows, Android, Linux, iOS, etc.) | 352 | 32.0 |
| Acquire ad-hoc knowledge in the field of web marketing (SEO, SEM, Web Analytics, Inbound Marketing, etc.) | 158 | 14.4 |
| Acquire knowledge of visual and graphic aspects of communication (with programs such as Photoshop, Lightroom, Illustrator, etc.) | 274 | 24.9 |
| Learn programming languages such as PHP, Java, SQL, HTML, etc. | 247 | 22.5 |
| Learning to create video games with various platforms | 98 | 8.9 |
| If you need help, would there be someone who could help you with using the Internet? | count | % |
| Yes | 719 | 65.4 |
| No | 120 | 10.9 |
| Maybe | 261 | 23.7 |
| Total | 1100 | 100.0 |
| Have you looked or asked for help to use the Internet in the past three months? | count | % |
| Yes | 267 | 24.3 |
| No | 807 | 73.4 |
| I don't remember | 26 | 2.4 |
| Total | 1100 | 100.0 |
| Have you helped someone use the Internet in the past three months? | count | % |
| Yes | 731 | 66.5 |
| No | 349 | 31.7 |
| I don't remember | 20 | 1.8 |
| Totale | 1100 | 100.0 |

Table 4. Request for help, formal training received, and help offered

**Descriptive statistics - Digital Competences**

| Please indicate how accurate the following statements are when thinking about how you use the Internet | Not at all true of me | Not very true of me | Neither true nor - untrue | Mostly true of me | Very true of me |
| --- | --- | --- | --- | --- | --- |
|  | **%** | **%** | **%** | **%** | **%** |
| I am confident in browsing, searching and filtering data, information and digital content | 3.3 | 13.6 | 23.0 | 45.0 | 13.8 |
| I regularly use cloud information storage services or external hard drives to save or store files or content | 13.2 | 11.8 | 17.9 | 35.7 | 19.7 |
| I regularly verify the sources of the information I find | 1.8 | 6.7 | 17.7 | 41.8 | 30.8 |
| I actively use a wide range of communication tools (e-mail, chat, SMS, instant messaging, blogs, micro-blogs, social networks) for online communication | 3.0 | 7.2 | 11.5 | 34.7 | 42.9 |
| I know when and which information I should and should not share online | 1.3 | 4.8 | 13.7 | 41.7 | 37.0 |
| I actively participate in online spaces and use several online services (e.g. public services, e-banking, online shopping) | 7.6 | 8.7 | 13.5 | 37.5 | 31.1 |
| I have developed strategies to address cyberbullying and identify inappropriate behaviours | 18.0 | 15.4 | 27.5 | 23.8 | 8.0 |
| I can produce complex digital content in different formats (e.g. images, audio files, text, tables) | 15.9 | 13.2 | 19.9 | 31.3 | 17.1 |
| I can apply advanced formatting functions of different tools (e.g. mail merge, merging documents of different formats) to the content I or others have produced | 17.8 | 15.1 | 22.3 | 26.6 | 15.9 |

Table 5. *Information and data literacy* and *Communication and collaboration*

| Please indicate how accurate the following statements are when thinking about how you use the Internet | Not at all true of me | Not very true of me | Neither true nor – untrue | Mostly true of me | | Very true of me |
| --- | --- | --- | --- | --- | --- | --- |
|  | **%** | **%** | **%** | **%** | | **%** |
| I respect copyright and licences rules and I know how to apply them to digital information and content | 3.6 | 6.3 | 19.6 | | 36.2 | 29.8 |
| I am able to apply advanced settings to some software and programs | 17.6 | 17.3 | 21.4 | | 27.5 | 13.1 |
| I periodically check my privacy setting and update my security programs (e.g. antivirus, firewall) on the device(s) that I use to access the Internet | 7.0 | 11.2 | 20.9 | | 38.2 | 20.8 |
| I use different passwords to access equipment, devices and digital services | 4.5 | 8.5 | 14.1 | | 36.1 | 34.8 |
| I am able to select safe and suitable digital media, which are efficient and cost-effective in comparison to others | 5.4 | 7,0 | 25.9 | | 39.3 | 19.2 |
| I am able to solve a technical problem or decide what to do when technology does not work | 11.9 | 13.6 | 20.7 | | 35.6 | 16.3 |
| I can use digital technologies (devices, applications, software or services) to solve (non-technical) problems | 8.6 | 11.6 | 21.8 | | 37.8 | 16.4 |
| I am able to use varied media to express myself creatively (text, images, audio and video) | 7.5 | 10.5 | 23.5 | | 37.5 | 18.7 |
| I frequently update my knowledge on the availability of digital tools | 9.6 | 13.3 | 25.1 | | 35.0 | 15.5 |

Table 6. *Digital content creation*, *Safety* and *Problem-solving*

| Variables |  | Italian Sample | UK Sample |
| --- | --- | --- | --- |
| Qualification | Some high school, no diploma | 43.0 | 64.0 |
|  | High school graduate | 50.7 | 70.0 |
|  | Some college credit, no degree | 51.2 | 73.9 |
|  | Bachelor's degree | 51.9 | 74.4 |
|  | Master's degree | 60.6 | 78.3 |
|  | Postgraduate qualification | 62.5 | 84.3 |
|  |  | F=13.692; Sig. <.000 | F=8.859; Sig.<.000. |
| Age | Correlation | -.404^**^ | −.397** |
| Gender | Male | 52.8 | 74.0 |
|  | Female | 49.2 | 71.6 |
|  |  | F=.025; Sig.= .873 | F=. 245; Sig.=. 621 |
| Income | Less than 10.000€ | 48.9 | 67.7 |
|  | 10.000€ - 20.000€ | 50.6 | 70.6 |
|  | 21.000€ - 30.000€ | 50.9 | 73.0 |
|  | 31.000€ - 50.000€ | 52.0 | 77.0 |
|  | More than 50.000€ | 55.2 | 81.6 |
|  |  | F=2.995; Sig. <.018 | F=5.347; Sig.=.000 |
| Area of residence | Urban areas | 55,1 | 75.2 |
|  | Small towns | 51,0 | 72.1 |
|  | Rural areas | 47.8 | 68.6 |
|  |  | F=18.919; Sig. <.000 | F=4.745; Sig. <.003 |

**Correlation is significant at the .01 level (two-tailed)

Table 7. Italian-UK comparison
